# Free Fatty Acid Induces Endoplasmic Reticulum Stress and Apoptosis of β-cells by Ca^2+^/Calpain-2 Pathways

**DOI:** 10.1371/journal.pone.0059921

**Published:** 2013-03-20

**Authors:** Wei Cui, Jie Ma, Xingqin Wang, Wenjuan Yang, Jing Zhang, Qiuhe Ji

**Affiliations:** 1 Department of Endocrinology and Metabolism, Xijing Hospital, Fourth Military Medical University, Xi’an, China; 2 Department of Neurosurgery, Tangdu Hospital, Forth Military Medical University, Xi’an, China; 3 No. 371 Central Hospital of People’s Liberation Army, Xinxiang, China; Thomas Jefferson University, United States of America

## Abstract

Dysfunction of β-cells is a major characteristic in the pathogenesis of type 2 diabetes mellitus (T2DM). The combination of obesity and T2DM is associated with elevated plasma free fatty acids (FFAs). However, molecular mechanisms linking FFAs to β-cell dysfunction remain poorly understood. In the present study, we identified that the major endoplasmic reticulum stress (ERS) marker, Grp78 and ERS-induced apoptotic factor, CHOP, were time-dependently increased by exposure of β-TC3 cells to FFA. The expression of ATF6 and the phosphorylation levels of PERK and IRE1, which trigger ERS signaling, markedly increased after FFA treatments. FFA treatments increased cell apoptosis by inducing ERS in β-TC3 cells. We also found that FFA-induced ERS was mediated by the store-operated Ca^2+^ entry through promoting the association of STIM1 and Orai1. Moreover, calpain-2 was required for FFA-induced expression of CHOP and activation of caspase-12 and caspase-3, thus promoting cell apoptosis in β-TC3 cells. Together, these results reveal pivotal roles for Ca^2+^/calpain-2 pathways in modulating FFA-induced β-TC3 cell ERS and apoptosis.

## Introduction

Type 2 diabetes mellitus (T2DM) is a multifactorial disease induced by genetic and many other environmental factors. Dysfunction of β-cells is a major characteristic in the pathogenesis of T2DM [1]. The prevalence of obesity in modern society has increased dramatically over the past few years and has reached epidemic proportions. Obesity, if sustained, may result in dyslipidemia, hypertension, glucose intolerance, insulin resistance and inflammation [2]. The combination of obesity and T2DM, is associated with excessive release of fatty acids from the expanded adipose tissue mass, leading to elevated plasma free fatty acids (FFAs) [3].

Dysfunction of β-cells is induced by several molecules including glucose, FFAs, and certain cytokines such as TNF-α [4]. Elevated plasma FFAs levels, which are accompanied by obesity, may play a causal role in β-cell dysfunction. It has been reported that acute FFA exposure stimulates insulin secretion, while prolonged FFA exposure decreases glucose-stimulated insulin secretion (GSIS) [5], [6]. However, molecular mechanisms linking FFA to β-cell dysfunction remain poorly understood.

The endoplasmic reticulum (ER) is responsible for protein folding and assembling into newly synthesized secretory proteins. When its function is disturbed by various physiological and pathological conditions such as misfolded protein accumulation, hypoxia, Ca^2+^ depletion, or microbial infection, endoplasmic reticulum stress (ERS) develops. ERS can regulate processes such as cell survival and cell death. Diets rich in saturated fats cause obesity. Concomitantly, cells are exposed to increased levels of FFAs of dietary origin or released by adipose tissues. Chronic exposure to high FFAs concentrations causes ERS which may contribute to cell apoptosis [2]. Growing evidence suggests that ERS signaling has been associated with Parkinson’s disease (PD), T2DM, and many other human diseases [7].

Unregulated Ca^2+^ influx might be a mediator of β-cell dysfunction and apoptosis in T2DM. In nonexcitable cells, such as β-cells, store-operated Ca^2+^ entry is the predominant Ca^2+^ influx mechanism [8]. Recent studies have identified that STIM1 and Orai1 are responsible for store-operated Ca^2+^ entry [9], [10]. Upon depletion of internal Ca^2+^ stores, STIM1, the endoplasmic reticulum-resident Ca^2+^ store sensor, translocate to areas near the plasma membrane to signal the activation of store-operated Ca^2+^ channels encoded by Orai1 proteins. Association of STIM1 with Orai1 is sufficient to reconstitute the store-operated Ca^2+^ channel function [9], [10]. Dysregulation of store-operated Ca^2+^ entry has been identified predominant incentive resulting in Ca^2+^ overload [11]. It is well recognized that cytoplasmic Ca^2+^ overload is a ubiquitous cause of cell death in neurons, cardiomyocytes, and insulin-producing β-cells [12], [13]. Effectors or executors of Ca^2+^ overload include calpains, kinases/phosphatases, calmodulin, and calcineurin [14]. Ca^2+^-dependent calpains belong to the cysteine protease family, and sustained hyperactivation of calpain is provoked in many pathological processes, including T2DM, ischemia, traumatic injury, and neurodegenerative disorders such as Alzheimer’s disease [13], [15], [16]. β-cells express several calpains, including calpain-10, calpain-1, and calpain-2. Polymorphisms in calpain-10 are associated with the risk of developing T2DM in some ethnic groups [17].

In this study, we found that by promoting store-operated Ca^2+^ entry through STIM1-Orai1 pathway, FFA treatments increase calpain-2 activity to induce expression of CHOP and activation of caspase-12 and caspase-3, thus enhancing cell apoptosis in β-TC3 cells.

## Results

### FFA Treatments Induce ERS in β-TC3 Cells and Increase Cell Apoptosis

**Figure 1:**
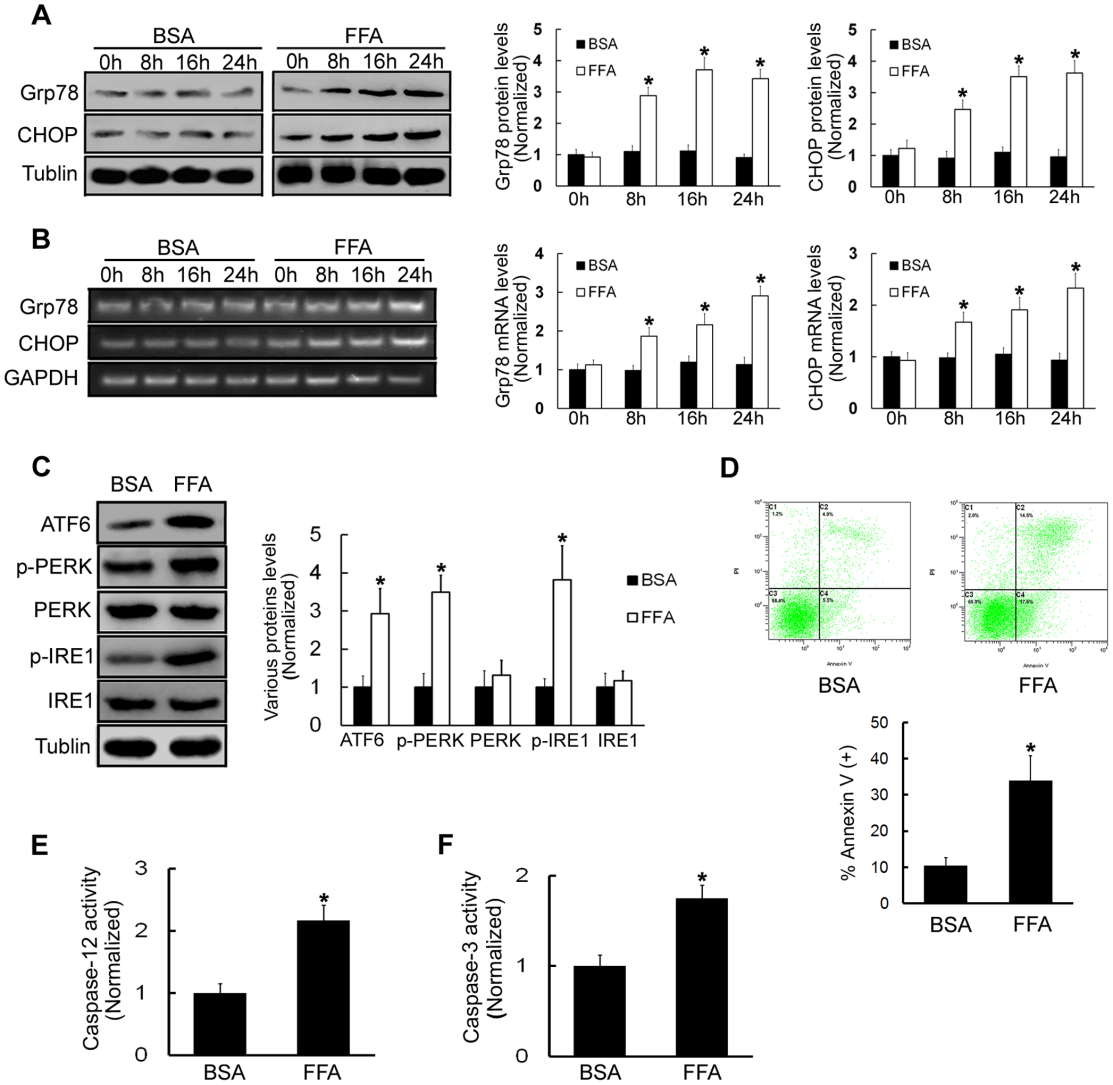
FFA treatments induce ERS in β-TC3 cells and increase cell apoptosis. (A) Cells were treated with 0.5 mM FFA or BSA for 0, 8, 16, or 24 h. Western blot was used to examine Grp78 and CHOP protein levels. (B) RT-PCR was used to test Grp78 and CHOP mRNA levels. (C) Cells were treated with 0.5 mM FFA or BSA for 16 h. Western blot was used to examine the expression levels of ATF6, p-PERK, PERK, p-IRE1 and IRE1. (D) Cells were treated with 0.5 mM FFA or BSA for 16 h. Cell death was quantified by annexin V/PI double staining. (E) Caspase-12 activity was detected after cells were treated with 0.5 mM FFA or BSA for 16 h. (F) Caspase-3 activity was detected after cells were treated with 0.5 mM FFA or BSA for 16 h. BSA-treated cells were used as a negative control. Bars represent each sample performed in triplicate, and the error bars represent the standard deviations. *P<0.05, by the Student’s *t*-test.

To examine whether FFA triggers ERS in β-TC3 cells, we examined the expression patterns of several molecular indicators of ERS. The ER chaperon glucose regulated 78-kDa protein (Grp78), also named Bip, and the transcription factor CHOP are central regulators of ERS. Mouse pancreatic β-TC3 cells were treated with FFA (0.5 mM) for different times (0, 8, 16, or 24 h). Bovine serum albumin (BSA) was used as a negative control. The results showed that Grp78 and CHOP protein levels were time-dependently increased in cells treated with FFA compared with corresponding BSA treated cells (*P*<0.05, Figure 1A). The mRNA levels of Grp78 and CHOP were also increased in a time-dependently manner in cells treated with FFA compared with corresponding BSA treated cells (*P*<0.05, Figure 1B). In order to further assess whether FFA treatments could induce ERS, we examined the expression and phosphorylation levels of ATF6, PERK, and IRE1, the three key factors that trigger ERS signaling in β-TC3 cells. We observed that the expression of ATF6 and the phosphorylation levels of PERK and IRE1 markedly increased after FFA treatments for 16 h (293.4%±65.2%, 349.3%±43.7% and 381.6%±89.5%, respectively, *P*<0.05, Figure 1C). All these data demonstrated that ERS can be activated by FFA treatments in β-TC3 cells.

ERS might be a mediator of cell death, thus β-TC3 cell death was examined by annexin V/propidium iodide (PI) double staining after FFA treatments. The results demonstrated that FFA treatments caused more cell apoptosis than BSA control treatments (33.9%±6.91% and 10.42%±2.15%, respectively, *P*<0.05, Figure 1D). It is known that activation of murine caspase-12 is associated with ERS-induced cell apoptosis [18]. Thus, we detected the activation of caspase-12. Compared with the activation in BSA-treated β-TC3 cells, caspase-12 activation in FFA-treated β-TC3 cells was significantly increased (216.7%±24.3%, *P*<0.05, Figure 1E). Caspase-3 (also named CPP32, apopain, and YAMA) has been identified as a key mediator of apoptosis in mammalian cells. We further assessed the activation of caspase-3 (cleaved caspase-3), and the results showed that FFA treatments increased caspase-3 activation (175.4%±14.2%, *P*<0.05, Figure 1F). From the above results, we concluded that FFA treatments could induce ERS and increase apoptosis in β-TC3 cells.

### FFA Treatments Promote Store-operated Ca^2+^ Entry to Induce ERS in β-TC3 Cells

**Figure 2:**
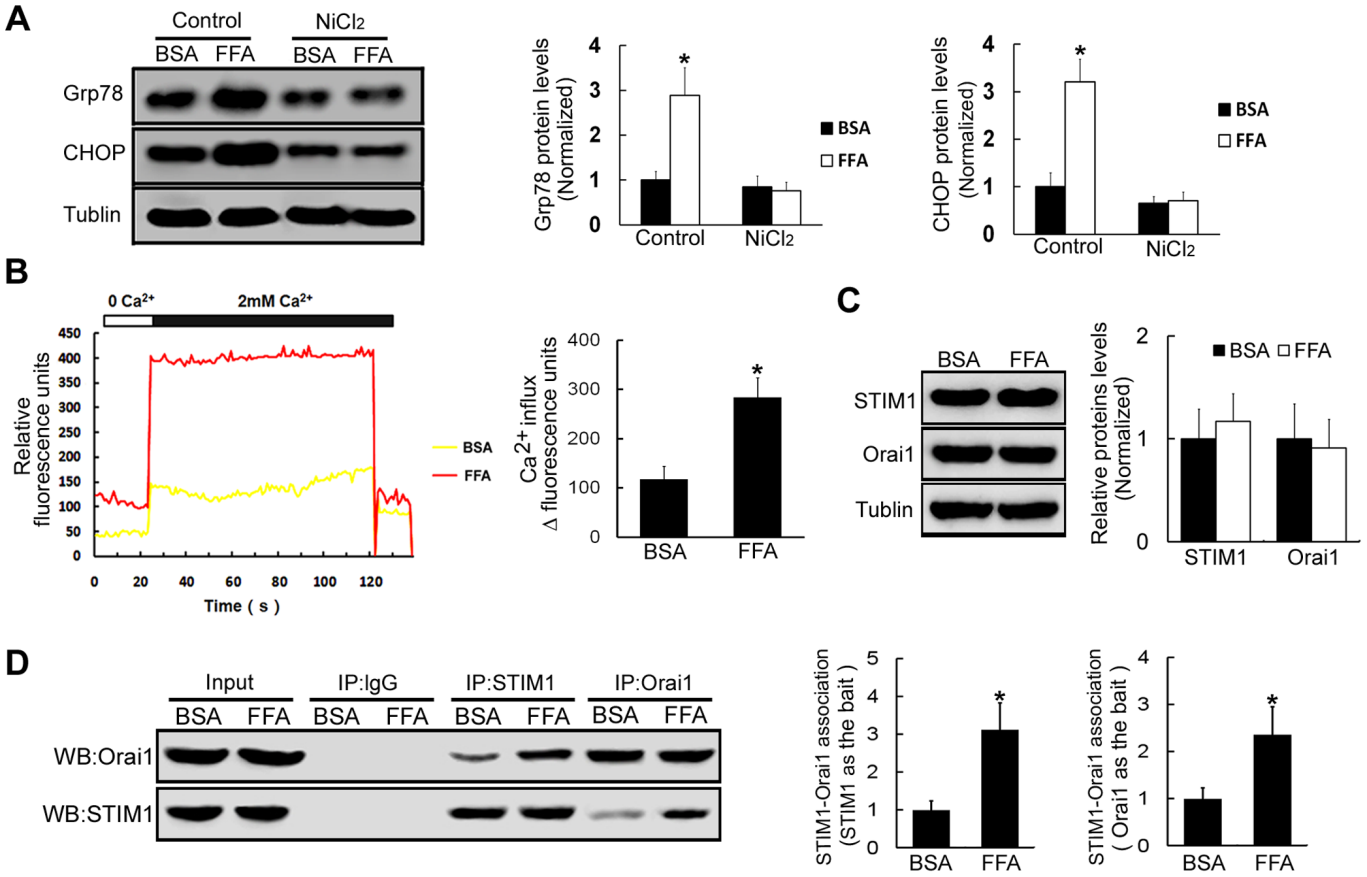
FFA treatments increase Ca^2+^ influx to induce ERS in β-TC3 cells. (A) β-TC3 cells were incubated with FFA or BSA for 16 h, western blot was used to examine the protein expression levels of Grp78 and CHOP following the treatment with Ca^2+^ channel blocker NiCl_2_. (B) β-TC3 cells were incubated with FFA or BSA for 16 h, and then stimulated with 4 µM thapsigargin for 20 min to activate store-operated Ca^2+^ entry. Fluorescence densities of Ca^2+^ change were monitored in Fluo-8/AM-loaded β-TC3 cells after FFA or BSA treatments. (C) The protein expression levels of STIM1 and Orai1 were tested by western blot following treatments with FFA or BSA for 16 h in β-TC3 cells. (D) β-TC3 cells were incubated with FFA or BSA for 16 h, and then stimulated with 4 µM thapsigargin for 20 min. Cell lysates were immunoprecipitated with anti-STIM1 antibody followed by western blot using anti-Orai1 antibody and with anti-Orai1 antibody followed by western blot using anti-STIM1 antibody. Immunoprecipitated with anti-IgG antibody was used as the negative control. Bars represent each sample performed in triplicate, and the error bars represent the standard deviations. *P<0.05, by the Student’s *t*-test.

It is well known that the disruption of intracellular Ca^2+^ homeostasis can disturb ER function and induce ERS. In nonexcitable cells, such as pancreatic β-cells, Ca^2+^ influx is the predominant mechanism to maintain intracellular Ca^2+^ homeostasis [19]. To assess whether FFA treatments-induced ERS is mediated by Ca^2+^ influx changes in β-TC3 cells, NiCl_2_, a Ca^2+^ channel blocker, was used. The results showed that FFA treatments were not able to increase Grp78 and CHOP levels in the condition that Ca^2+^ influx was blocked with NiCl_2_ treatments (*P*>0.05, Figure 2A). The results suggested that FFA treatments induced-ERS is mediated by the increase of Ca^2+^ influx. One of the major routes for Ca^2+^ influx into cells is through store-operated Ca^2+^ entry channels [8]. To further assess whether FFA treatments may induce store-operated Ca^2+^ entry in β-TC3 cells, store-operated Ca^2+^ entry was examined after FFA treatments. The results demonstrated that FFA treatments resulted in more than a 2-fold increase of store-operated Ca^2+^ entry in β-TC3 cells compared with BSA treatments (*P*<0.05, Figure 2B). Recent studies have identified that STIM1 and Orai1 are responsible for store-operated Ca^2+^ entry. We found that the expression levels of STIM1 and Orai1 in cells treated with FFA was similar to cells treated with BSA (*P>*0.05, Figure 2C). However, immunoprecipitation with anti-STIM1 antibody followed by western blot with anti-Orai1 antibody revealed that the association of STIM1 with Orai1 in samples from FFA-treated cells, was much more than the association observed in BSA-treated cells (312.5%±71.4%, *P*<0.05, Figure 2D). The results were further strengthened with Orai1 as the bait (236.2%±59.1%, *P*<0.05, Figure 2D). The results suggested that FFA enhanced the association of STIM1 with Orai1. Therefore, these results indicated that FFA treatments induced-ERS is mediated by the store-operated Ca^2+^ entry which is regulated by the association of STIM1 with Orai1.

### By Increasing Calpain-2 Activity, FFA Treatments Promote CHOP Induction in β-TC3 Cells

**Figure 3:**
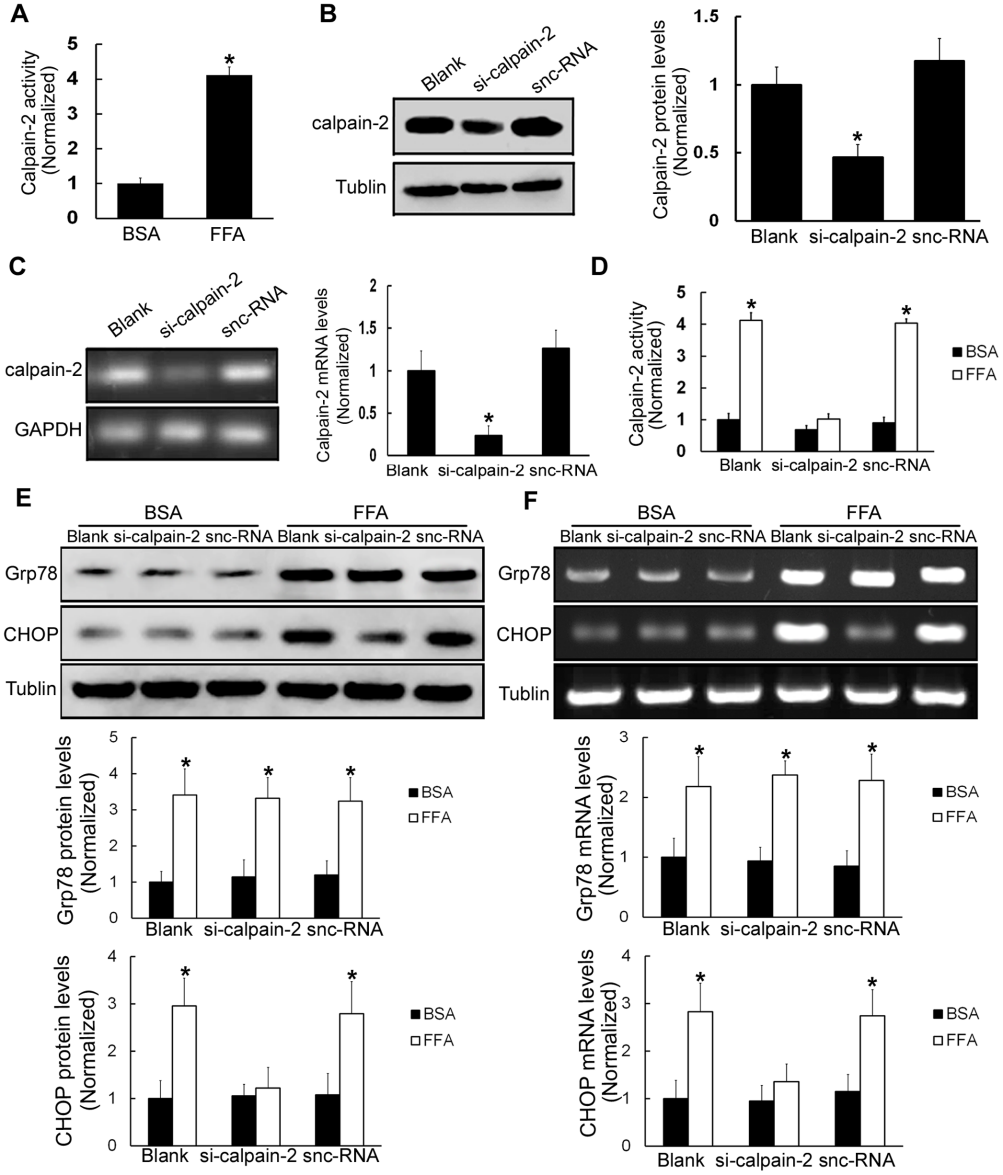
FFA treatments increase calpain-2 activity, thus inducing ERS in β-TC3 cells. (A) The activity of calpain-2 was tested in β-TC3 cells treated with 0.5 mM FFA or BSA for 16 h. (B) β-TC3 cells were treated with calpain-2 siRNA for 24 h. Western blot was performed to examine calpain-2 protein levels. (C) RT-PCR was used to analyze calpain-2 mRNA levels. (D) Calpain Activity Assay Kit was used to analyze calpain-2 activity. Silencer negative control siRNA (snc-RNA)-treated cells were used as a negative control. (E) β-TC3 cells were treated with calpain-2 siRNA for 24 h and then stimulated with FFA for 16 h, and western blot was used to examine the protein expression levels of Grp78 and CHOP. (F) RT-PCR was used to test Grp78 and CHOP mRNA levels. BSA-treated β-TC3 cells were used as a negative control. Bars represent each sample performed in triplicate, and the error bars represent the standard deviations. *P<0.05, by the Student’s *t*-test.

Calpain-2, a neutral Ca^2+^-dependent protease, mediates a variety of physiological functions such as cytoskeleton remodeling, vesicle trafficking, and membrane fusion [20]. To assess whether FFA treatments may influence calpain-2 activity in β-TC3 cells, calpain-2 activity was examined after FFA treatments. The results demonstrated that after FFA treatments, calpain-2 activity was dramatically enhanced in β-TC3 cells (411.4%±23.5%, *P*<0.05, Figure 3A). To examine whether FFA-induced ERS is dependent on calpain-2, we used small interfering RNA (siRNA) to knock down calpain-2 in β-TC3 cells. Calpain-2 siRNA led to a marked reduction of calpain-2 protein and mRNA levels (46.7%±9.5% and 23.4%±11.6%, respectively, *P*<0.05, Figure 3B and Figure 3C). When the cells were treated with calpain-2 siRNA, FFA treatments did not increase calpain-2 activity compared with corresponding BSA treatments in β-TC3 cells (*P*>0.05, Figure 3D). To further validate whether calpain-2 is involve in FFA-triggered ERS in β-TC3 cells, we examined the protein and mRNA levels of Grp78 and CHOP in the condition that calpain-2 activity was inhibited with calpain-2 siRNA. The results showed that FFA treatments increased Grp78 protein and mRNA levels following calpain-2 siRNA treatments (*P*<0.05, Figure 3E and Figure 3F). However, FFA treatments were not able to increase CHOP protein and mRNA levels following calpain-2 siRNA treatments (*P*>0.05, Figure 3E and Figure 3F). These results suggested that calpain-2 is involved in FFA-induced CHOP expression, but not involved in FFA-induced Grp78 expression.

### Calpain-2 is Required for FFA-induced β-TC3 Cell Apoptosis

**Figure 4:**
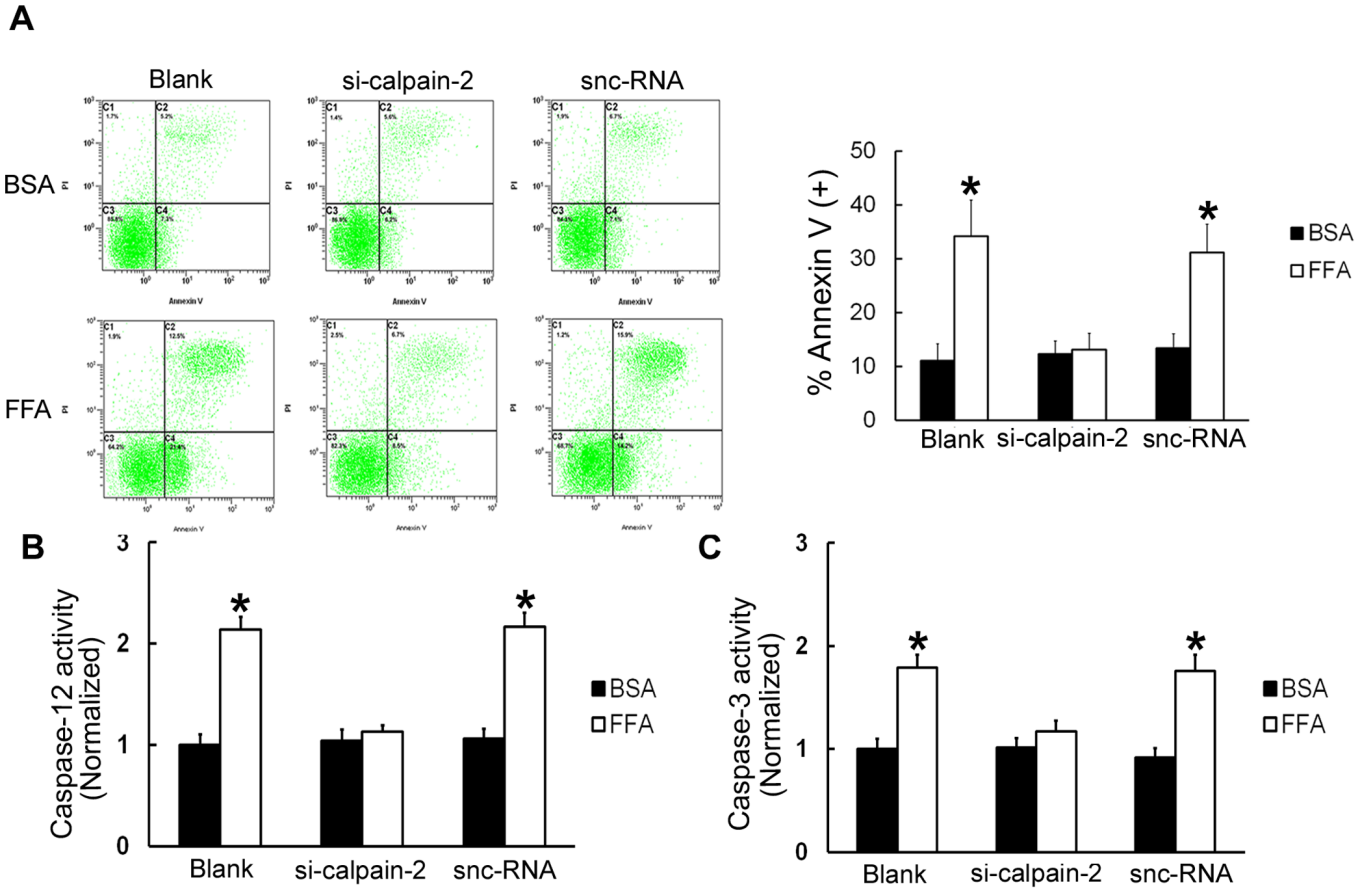
Calpain-2 is required for FFA treatment-induced β-TC3 cell apoptosis. The cells were treated with calpain-2 siRNA, incubated for 24 h, and then stimulated with FFA or BSA for 16 h. (A) Cell death was quantified by annexin V/PI double staining. (B) Caspase-12 activity was detected after cells were treated with calpain-2 siRNA. (C) Caspase-3 activity was detected after cells were treated with calpain-2 siRNA. snc-RNA-treated cells were used as a negative control. Bars represent each sample performed in triplicate, and the error bars represent the standard deviations. *P<0.05, by one-way ANOVA.

The above results demonstrated that FFA treatments can increase apoptosis in β-TC3 cells. To further confirm that calpain-2 is involved in this process, calpain-2 siRNA was used. Annexin V/PI double staining demonstrated that FFA treatments were not able to increase the percentage of apoptotic cells compared with corresponding BSA treatments following calpain-2 siRNA treatments (*P*>0.05, Figure 4A). To corroborate these results, the activation of caspase-12 and caspase-3 were detected. The results showed that FFA treatments were not able to increase caspase-12 and caspase-3 activation compared with corresponding BSA treatments following calpain-2 depletion (*P*>0.05, Figure 4B and Figure 4C). Therefore, we concluded that calpain-2 is required for FFA-induced β-TC3 cell apoptosis.

## Discussion

This study was designed to identify the molecular mechanisms of β-cell dysfunction induced by FFA. The underlying concept is that once the primary pathogenesis of diabetes is established, hyperglycemia and very commonly hyperlipidemia ensue, and thereafter they exert additional damaging or toxic effects on β-cells [21]. It has been suggested that insulin resistance precedes the development of T2DM. A common feature of insulin resistant states is high plasma FFA content [22]. In addition to reducing insulin sensitivity in peripheral tissues, elevated plasma FFA levels may also contribute to the deterioration of β-cell function. Many studies, using insulin-secreting cells and isolated islets, have attempted to identify the mechanisms of lipotoxicity in β-cell dysfunction. In vitro, prolonged exposure of isolated islets or insulin-secreting cells to elevated FFA levels is associated with inhibition of GSIS [23], [24], which has also been observed in vivo in rats [25] and humans [26]; impairment of insulin gene expression [27]–[32]; and induction of cell apoptosis [33], [34]. In vitro, prolonged exposure of isolated islets or insulin-secreting cells to elevated FFA causes β-cell apoptosis which may contribute to the loss of β-cell mass in T2DM [23], [24]. Moreover, ERS has been reported to link FFA–induced β-cell apoptosis with insufficient insulin secretion in rodents. Previous researches indicated that excessive ERS can trigger cellular apoptosis through the activation of caspase-12 [29]–[32]. Furthermore, CHOP, a crucial transcription factor in ERS induced apoptosis, mediates cell death through the induction of various genes including GADD34 and ERO1, which may tip the balance towards apoptosis [33], [34]. However, the detailed mechanism that FFA-triggered β-cell apoptosis and eventually enhanced loss of β-cell mass in T2DM has not been determined. Our data indicated that FFA could induce ERS through enhancing Ca^2+^ influx which is mediated by promoting the association of STIM1 and Orai1. Furthermore, we showed that FFA could activate calpain-2 to induce CHOP expression and β-cells apoptosis, thus enhancing loss of β-cell mass in T2DM.

We demonstrated that the expression levels of molecular indicators of ERS, Grp78 and CHOP, are time-dependently increased by exposure of β-cells to FFA. The expression of ATF6 and the phosphorylation levels of PERK and IRE1, which trigger ERS signaling, markedly increase after FFA treatments. These results demonstrated that ERS could be activated by FFA treatments in β-cells. ERS has been demonstrated to be involved in apoptosis induction during pathophysiological processes, including diabetes [35]. Grp78, the major ER-resident chaperone and the most abundant glycoprotein in the ER, plays critical roles in protein folding and ER Ca^2+^ binding, and it is widely used as a biomarker of ERS [36]. Moreover, other ERS-related chaperones, such as CHOP, are also components of the apoptosis pathway mediated by ERS [37]. In mammalian cells, upon ERS, the ER transmembrane kinase IRE1 initiates the splice processing of XBP1 mRNA and results in activation of XBP1, which can then bind ERS response elements and activate the transcriptional set of ER chaperones, such as Grp78 and CHOP [38]. Several studies have shown that the upregulation of these ERS-related chaperones is believed to induce ERS, leading to cell apoptosis by toxic insults [39].

Aberrantly increased cytoplasmic Ca^2+^ has been shown to mediate cell dysfunction and cell death in neurodegenerative diseases [40]. Store-operated Ca^2+^ entry (SOCE) is a common and ubiquitous mechanism of regulating Ca^2+^ influx into cells [10]. Two genes, STIM1 and Orai1, are responsible for store-operated Ca^2+^ entry activation. Once endoplasmic reticulum Ca^2+^ depleted, STIM1 proteins aggregate into multiple puncta and translocate to the plasma membranes. Orai1 molecule, an essential component of the SOCE channel, translocates to the same STIM1-containing structures to form association with STIM1 during store depletion and opens to mediate Ca^2+^ entry [9], [10]. Our studies implied that the enhancement of store-operated Ca^2+^ entry which is regulated by the association of STIM1 with Orai1 contributed to the cytotoxicity of FFA. Moreover, these data suggested that dysfunction and increased apoptosis of β-cells are mediated, at least partially, through FFA induced Ca^2+^ overload which caused by association of STIM1 with Orai1.

The calpains are a family of Ca^2+^-dependent, non-lysosomal, neutral cysteine endopeptidases [41]. They are known to play important roles in several Ca^2+^ regulated physiological processes, including cytoskeletal remodeling, cellular signaling, cell proliferation, cell cycle progression, apoptosis, and cell survival [42]. Two isoforms in particular, calpain-1 (μ-calpain) and calpain-2 (m-calpain), have been extensively studied. Both isoforms function as heterodimeric enzymes and are composed of a distinct, large catalytic subunit (80 kDa) associated with a common, small regulatory subunit (30 kDa) that helps maintain calpain activity [43], [44]. Calpain activity is regulated by multiple mechanisms, including Ca^2+^ modulation, autoproteolysis, phosphorylation, intracellular distribution, and inhibition by calpastatin. The best studied mechanism is Ca^2+^ activation, because calpains contain Ca^2+^ binding EF-hand motifs in domains IV and VI [41]. The two isoforms differ not only in their amino acid sequence of the large catalytic subunits, but also in their Ca^2+^ requirements for activation in vitro. Calpain-2 requires millimolar concentrations of Ca^2+^, whereas calpain-1 requires micromolar concentrations. In general, however, these concentrations of Ca^2+^ are far greater than that can be achieved intracellularly, suggesting that additional factors, such as phospholipids or activation proteins, are required for activation of both isoforms in vivo [45]. In the study, we found that calpain-2 is markedly activated and plays critical roles in FFA induced cell apoptosis. CHOP, known as growth arrest and DNA damage inducible gene, is a member of the C/EBP transcription factor family. It has been reported that CHOP induction, which occurs during several responses to cellular stress, is involved in the ERS-induced apoptosis pathway [37]. In particular, CHOP is the first molecule identified to mediate ERS-induced apoptosis. CHOP inhibits expression of Bcl-2, depletes glutathione, facilitates translocation of proapoptotic protein Bax, and thereby induces apoptosis [46]. We observed that inhibition of calpain-2 decreased CHOP expression after FFA treatments. Thus, calpain-2-induced CHOP expression may result in FFA treatments-induced β-cells apoptosis. It is reported that transcription factor ATF2 may bind to CHOP promoter to promote CHOP expression. The transactivating capacity of ATF2 is depending on phosphorylation of N-terminal residues Thr69, Thr71, and Ser90 [47], [48]. We suspect that by phosphorylation of ATF2, calpain-2 affects the expression of CHOP. The study is underway in our lab.

Most ERS-related pro-apoptotic signals ultimately lead to caspase activation. Caspase-12 and caspases-3, which have been proposed as specific mediators of ERS-induced apoptosis in rodent cells [49], were observed activated after FFA treatments in our study. Furthermore, we found that calpain-2 is required for FFA treatments-induced cell apoptosis and activation of caspase-12 and caspase-3 in β-cells, which may account for cell apoptosis in hyperlipidemia induced T2DM. Therefore, the inhibition of calpain-2 may decrease hyperlipidemia-induced β-cells apoptosis in T2DM. Given the nature of in vitro study, our work has some limitations. The extensive interactions among cells and tissues cannot be completely duplicated in vitro study. Our results should be further demonstrated in animal models, and the study is underway in our lab.

The contribution of Ca^2+^ to FFA treatments-induced ERS in β-cells has been established for many years, but as Ca^2+^ is a widely existed second messenger,which has been shown to play roles in the regulation of proliferation, invasion, and metastatic potential, the side effect of inhibition Ca^2+^ level is major challenges to its clinical application. As we demonstrated that calpain-2 is required for FFA treatments-induced ERS in β-cells. The development of new therapeutic approaches, including small molecules or antibodies that can specifically block calpain-2 expression and/or its activity, is clinically feasible in the future. In this regard, drugs that target calpain-2 may represent a novel approach to T2DM treatment.

## Materials and Methods

### Cell Culture

Mouse β-TC3 cell line, which was kindly provided by Novo Nordisk, Copenhagen, Denmark [50], was cultured in RPMI 1640 medium (Gibco, Grand Island, USA), supplemented with 10% FBS, 1% penicillin/streptomycin, and 2% L-glutamine at 37°C in a humidified atmosphere of 5% CO_2_.

### Treatment of Cells with FFA

β-TC3 cells were treated with 0.5 mM palmitic acid (Sigma, St. Louis, MO, USA) for 0, 8, 16, and 24 h. Equivalent amounts of fatty acid-free BSA were added to the control group. Palmitic acid solution was prepared as described previously [51].

### Gene Silencing

The sense sequence for calpain-2 siRNA was 5′-GCGAGGACATGCACACCAT-3′ (GenePharma, China). The cells were transfected with siRNA using the LipofectAMINE 2000 reagent (Invitrogen, CA, USA) according to the manufacturer’s instructions. The sense sequence for silencer negative control siRNA (snc-RNA) was 5′-AGTCGACGTCAGCTGAAGGC-3′ (GenePharma, China), which was used as a negative control under similar conditions.

### Western Blot Analysis

Cells were lysed in 1% OG buffer (20 mM Tris-HCl (pH 8.0), 150 mM NaCl, 1% OG, 1 mM EDTA, 10 µg/ml leupeptin, 2 µg/ml aprotinin, and 1 mM PMSF). BCA Protein Assay Kit (Pierce Biotechnology, Rockford, IL, USA) was then used to determine the total protein density. Then equal amounts of protein were separated by 10% SDS-PAGE and transferred to a polyvinylidene fluoride (PVDF) microporous membrane (Millipore Billerica, MA, USA). After being blocked with 5% non-fat milk, the membrane was incubated for 2 h at room temperature with the designated antibody. A Western-Light chemiluminescent detection system (Applied Biosystems, Foster City, CA, USA) was used for immunodetection.

### Immunoprecipitation Analysis

β-TC3 cells were preincubated with 0.5 mM FFA or fatty acid-free BSA for 16 h before treated with 4 µM thapsigargin (Invitrogen, CA, USA) for 20 min to activate store-operated Ca^2+^ entry. Cells were then washed and resuspended in NPBS. Cells were lysed in 1% OG buffer. BCA Protein Assay Kit (Pierce Biotechnology, Rockford, IL, USA) was then used to determine the total protein density. Fraction of the lysates was saved as “input”. Then, aliquots of lysates (1 mL) were immunoprecipitated by 25 µL of protein A-agarose that was pre-bound with 2 µg of anti-IgG antibody, anti-STIM1 antibody or anti-Orai1 antibody overnight at 4°C. Bound proteins were then eluted from protein A-agarose with elution buffer, and aliquots of the eluent were submitted to western blot using anti-Orai1 antibody diluted 1∶250 in TBST or anti-STIM1 antibody diluted 1∶300 in TBST for 2 h at room temperature. A Western-Light chemiluminescent detection system (Applied Biosystems, Foster City, CA, USA) was used for immunodetection.

### Reverse Transcriptase Polymerase Chain Reaction (RT-PCR)

Total RNA was extracted from the cells with TRIzol reagents (Invitrogen) and reversely transcribed into cDNA with the ReverTra Ace-a kit (Toyobo). The primers were synthesized by Shanghai Sangon Co. as follows: Grp78, forward primer 5′-CACAGACGGGTCATTCC-3′, reverse primer 5′-CCTATGTCGCCTTCACT-3′; CHOP, forward primer 5′-GACGCTTCACTACTCTTGACCCTGCG-3′, reverse primer 5′-GGATGTGCGTGTGACCTCTGT-3′; calpain-2, forward primer 5′-GCAGCCATTGCCTCCCTCAC-3′, reverse primer 5′-ACCTCCACCCACTCGCCGTA-3′; GAPDH, forward primer 5′-AATGTCACCGTTGTCCAGTTGC-3′, reverse primer 5′-CACCATCTTAGGAGGAGGAGTAGC-3′. GAPDH mRNA was used as an internal control. The PCR conditions were 1 cycle of 94°C for 5 min; 35 cycles of 94°C for 60 s, 57°C for 30 s, and 72°C for 30 s; and finally 72°C for 5 min. PCR products were electrophoresed on 1% agarose gels.

### Measurement of Store-operated Ca^2+^ Entry

Store-operated Ca^2+^ entry was measured using Fluo-8 acetoxymethyl ester(Fluo-8/AM)(Abcam, Cambridge, UK). β-TC3 cells were preincubated with FFA or BSA for 16 h before loading with the dye by incubation with 0.1 mg/ml Fluo-8/AM for 45 min in the dark at 37°C. Cells were then washed and resuspended in NPBS. To start the experiment, cells were pretreated with 4 µM thapsigargin (Invitrogen, CA, USA) for 20 min to deplete internal Ca^2+^ stores. After washing off extracellular thapsigargin, Ca^2+^-free phosphate-buffered saline (PBS) containing 3 mM EGTA was then added to the cells. To record Ca^2+^ influx, 2 mM Ca^2+^ was added after approximately 20 s. Ca^2+^ influx levels were recorded from Fluo-8 loaded cells excited at wavelengths of 340 nm and 380 nm and imaged with 510 nm filters. The fluorescence signal was monitored and recorded by an FV300 laser scanning confocal microscope (Olympus).

### Measurement of Calpain-2 Activity

Calpain-2 activity was determined using the Calpain Activity Assay Kit (Abcam, Cambridge, UK) following the manufacturer’s instructions. In brief, cell lysates were incubated at 37°C with Ac-LLY-AFC, a calpain-2 substrate, for 1 h. The fluorescence of the cleaved substrate was measured by a spectrofluorometer (Molecular devices, CA, USA).

### Annexin V/PI Double Staining

The number of dead cells was determined by annexin V/PI double staining. Cells were exposed to 0.5 mM FFA or BSA for 16 h and then incubated with FITC-conjugated annexin V in binding buffer (0.01 µM HEPES, 0.14 µM NaCl, and 2.5 mM CaCl_2_, pH 7.4) for 20 min at 37°C in the dark. After incubation, the cells were washed and resuspended in 200 µl PBS with 1% FCS and additionally incubated with 10 µl of 1 mg/ml PI solution. The annexin V-positive cells were detected using a FACSCalibur flow cytometer (BD Biosciences, San Jose, CA, USA), and the results were analyzed using CellQuest software (BD Biosciences, San Jose, CA, USA). Annexin V-FITC conjugates were detected with the FL1 channel of the FACSCalibur machine. PI was read on the FL2 channel.

### Detection of Caspase Activity

Caspase-3 activity was measured spectrophotometrically via the detection of pNA cleavage by caspase-3-specific substrates. These experiments were completed using a Caspase-3 Assay Kit (Beyotime, Shanghai, China). After the cell lysates were incubated with Ac-DEVD-pNA for 2 h at 37°C, the samples were read at 405 nm. Caspase-12 activity was measured spectrophotometrically via the detection of free AFC cleavage by caspase-12-specific substrates. These experiments were completed using a Caspase-12 Assay Kit (Biovision, San Francisco, CA, USA). After the cell lysates were incubated with ATAD-AFC for 2 h at 37°C, the samples were read at 505 nm.

### Statistical Analysis

Statistical analysis was performed using SPSS 13.0 statistical software. The results were expressed as mean values ± SD. And the Student’s t-test or one-way ANOVA were used to evaluate the statistical significance in the groups. The differences were considered significant when *P*<0.05.
